# An analysis of trunk kinematics and gait parameters in people with stroke

**DOI:** 10.4102/ajod.v7i0.310

**Published:** 2018-03-29

**Authors:** Adnil W. Titus, Susan Hillier, Quinette A. Louw, Gakeemah Inglis-Jassiem

**Affiliations:** 1 Department of Interdisciplinary Health Sciences, Stellenbosch University, South Africa; 2 Sansom Institute For Health Research, University of South Australia, Australia

## Abstract

**Background:**

Approximately two out of three people with stroke experience gait problems. Trunk movement control and symmetry is an important prerequisite for functional walking gait. Movement control, measured objectively as kinematics during walking gait, is rarely investigated.

**Objective:**

To describe the three-dimensional (3D) kinematics of the trunk during gait in people with stroke, including key spatiotemporal characteristics.

**Methodology:**

A total of 17 adults with stroke who met the inclusion criteria were selected to participate in this cross-sectional pilot study. An eight-camera T-10 Vicon system with Nexus 1.8 software (Vicon Motion System Limited, Oxford, UK) was used to analyse the 3D kinematics of the trunk during self-selected walking speed. Trunk kinematics throughout the gait cycle and spatiotemporal parameters were extracted using custom-built scripts in MATLAB used at the Stellenbosch University Movement Analysis Laboratory. Stata Version 12.1 software was used to assess differences in trunk kinematics between the affected and unaffected sides during gait using the Sign test (statistical significance level *p* < 0.05).

**Results:**

Participants achieved functional gait speeds although they presented with asymmetrical trunk kinematics. During the full gait cycle, there were statistically significant differences of trunk motion between the affected and unaffected sides in the coronal plane (*p* < 0.001). There were statistically significant differences in the trunk kinematics between the affected side and unaffected sides at initial contact (*p* < 0.001) and foot off (*p* < 0.049) in the coronal plane as well as at initial contact (*p* < 0.000) and foot off (*p* < 0.013) in the transverse plane.

**Conclusion:**

This pilot study found significant asymmetry in trunk motion between the affected and unaffected sides that varied across the gait cycle. This suggests the trunk may need to be targeted in clinical gait retraining post-stroke.

## Introduction

Stroke is a major global health concern in terms of mortality and chronic disability (Wissel et al. 2013). The global incidence of stroke is reported to account for approximately 5.5 million deaths annually and for 44 million disability-adjusted life-years lost (Mukherjee & Patil 2011). Hemiparesis is seen as the most common impairment after stroke and has a direct negative influence on the ability of a person to walk (Belda-Lois et al. 2011). Two out of three people experience persistent walking difficulties following a stroke (Stanhope et al. 2014). Walking difficulties in people with stroke are reported to be because of weakness (paresis) or abnormal tone of the limbs and trunk, impaired sensorimotor systems and central control mechanisms (Karthikbabu et al. 2011). The post-stroke gait pattern is characterised as hemiparetic with the predominant sensorimotor impairments being experienced in the contralesional upper and lower limbs. The role of the trunk in mobility and stability is however often overlooked as an integral component of performing daily core functions such as walking after stroke (Ryerson et al. 2008).

The term ‘trunk’ refers to the area between the midpoint of the hip joint centres caudally and the midpoint between the shoulder joint centres cranially (De Leva 1996). Trunk control is an essential component of functional walking gait (Carmo et al. 2012; Cromwell et al. 2001; Karthikbabu et al. 2011). It is defined as the ability of the muscles of the trunk to maintain an upright or neutral position, shift weight and selectively move to maintain the centre of gravity over the base of support (Karthikbabu et al. 2011). The muscles of the trunk actively contribute to balance during functional activities (Ceccato et al. 2009).

In healthy individuals, the trunk is maintained in a relatively neutral orientation, with negligible excursions in the sagittal, coronal and transverse planes during gait (Krebs et al. 1992). However, it has been reported that gait-related joint kinematics are generally different for people with hemiparesis compared to healthy people (Balaban & Tok 2014). Earlier kinematic research placed an emphasis on the pelvis and its role in gait, and not on the trunk segments above the pelvis. For example, Dodd and Morris (2003) specifically assessed the lateral pelvic displacement during gait of people with hemiparesis. Tyson (1999) reported on lateral translation of the trunk, but not on the remaining two planes (for rotation and flexion or extension). Balaban and Tok (2014) suggested that there is an increase in lateral trunk sway and elevation of the hip to allow for improved foot clearance in people with stroke. There is also an inference of rotation in that during gait the upper limb swings forward as the contralateral leg moves forward, and vice versa (Hacmon et al. 2012). Hacmon et al. (2012) and Verheyden et al. (2006) reported that people with stroke have weaker trunk muscles compared to their peers without stroke. These authors suggested that the trunk can be seen as a predictor of achieving walking ability post stroke rehabilitation. However, there is little objective information about trunk impairments during gait post-stroke.

Currently there is anecdotal evidence about impaired trunk control or movement during walking gait post-stroke in individuals who had a stroke. To inform rehabilitation strategies, empirical information is needed, which has also been identified by other researchers (Frigo & Crenna 2009). This study aimed to provide an objective evaluation of three-dimensional (3D) trunk kinematics during gait.

## Methodology

### Sample

In South Africa, individuals with stroke are referred to the community health centres for rehabilitation on an outpatient basis once they are medically stable. Seventeen participants, nine female and eight male, consented to participate in the study. Five male and five female participants had right hemiparesis and three male and four female participants had left hemiparesis. All the participants were recruited from a community health centre by means of convenience sampling. The inclusion criteria to participate in the study were as follows: men and women of 18 years and older, first ever confirmed stroke, ability to follow simple instructions and the ability to walk 10 m without assistive devices. People with bilateral signs, orthopaedic or other neurological pathologies that influence gait and any known allergies to the adhesive tape used during testing procedures were excluded. The mean age of the participants was 56.3 ± 9.5 (range 30–67 years), with the age at incidence being 51.8 ± 9.8 (range 27–67 years); mean time since stroke was 21 ± 18.0 months (range 2–51 months); and mean body mass index (BMI) for the group was 25.66 ± 4.24 (range 17.10–33.52).

### Setting

The study was conducted at the 3D Movement Analysis Laboratory of Stellenbosch University, which uses an eight-camera T-10 Vicon system (Vicon Motion System Ltd, Oxford, UK) with Nexus 1.8 software. The associated Vicon Plug-in-Gait (PiG) model was used to capture the 3D motion of the participants during walking at a self-selected comfortable speed.

### Procedure

Twenty-two retroreflective markers (14 mm diameter) were placed on participants’ bony landmarks according to the PiG model (lower limb markers were placed on the anterior and posterior superior iliac spines, lateral knee, lateral malleolus, second metatarsal head, heel, lateral thigh and tibia). The Vicon Motion Analysis system is regarded as the gold standard in 3D movement analysis because of its good reliability and validity (McGinley et al. 2009).

The PiG model offers a standardised procedure for the identification and placement of 22 body markers. Anthropometric measurements, including height, weight, leg length and knee and ankle width, were taken by an experienced laboratory technician.

The PiG model defines the trunk in three dimensions using Cardan angles. The Z-axis points downwards (longitudinal axis) and is perpendicular to the transverse plane, calculated from the midpoint between cervical spinous process 7 (C7) and the sternal notch (CLAV) to the midpoint of thoracic spinous process 10 (T10) and xiphoid process of the sternum (STRN). The X-axis points forward (sagittal axis) and is calculated from the midpoint between C7 and T10 to the midpoint between CLAV and STRN; it is perpendicular to the coronal plane. The Y-axis (coronal or transverse axis) points right, perpendicular to the X and Z axes, and runs perpendicular to the sagittal plane (Vicon 2010).

Anterior and posterior movement of the trunk (sagittal plane) refers to the trunk rotating latero-laterally, resulting in the anterior and posterior movements (flexion and extension) or tilting (Struyf et al. 2011). In the coronal plane during gait, Ceccato et al. (2009) describe the lateral movement (obliquity) of the trunk as a sideways curvature to the last swinging leg, assuming that this leg is now in the stance phase. Trunk rotation (transverse plane) is antiphase to the motion of the pelvis (Bruijn et al. 2008).

System calibration was performed as per the standard Vicon guidelines (Vicon 2010). Individual calibration was performed for each participant before they commenced walking using a static pose trial.

Participants were instructed to walk at a self-selected, comfortable speed along a 10 m distance of an even 30 m surface in the laboratory setting for a total of six trials, wearing the shoes they wore on the day of data capturing. The participants were allowed two practice trials. An average of all the shod trials was analysed and described in this paper. A stool was placed at either end of the walkway length for participants to rest if needed.

### Data processing

Preliminary marker reconstruction and labelling were performed using standard Vicon Nexus operations. Gap filling was performed using the standard Woltring filter supplied by Vicon. Specific points during the gait cycle were calculated, in degrees, using marker trajectories that correlated with gait phases. Trunk kinematics in the three different planes and spatiotemporal parameters were analysed in MATLAB (Mathworks, Natick, MA) using custom-built scripts.

### Statistical analysis

Descriptive statistics were calculated for spatiotemporal gait parameters and for trunk kinematics with mean and standard deviations in the three different planes. The mean and standard deviations of the kinematics were produced. Stata software was used to calculate the differences between the two sides (affected and unaffected) using the Sign test (statistical significance level *p* < 0.05).

## Results

### Spatiotemporal gait parameters

Table 1 summarises the averages of the spatiotemporal parameters including walking speed, cadence, step length, stride length, step time and stride time.

**TABLE 1:** Mean and standard deviation group spatiotemporal parameters.

Spatiotemporal parameters	Mean	SD	Max	Min	Range
Walking speed (m/s)	0.91	0.24	1.47	0.40	1.07
Cadence (steps/min)	101.63	16.21	130.00	67.00	63.00
Step length (m)	0.55	0.09	0.73	0.33	0.14
Stride length (m)	1.07	0.19	1.38	0.65	0.73
Step time (s)	0.61	0.10	0.90	0.46	0.44
Stride time (s)	1.21	0.17	1.70	0.94	0.76

*Source*: Authors’ own work

SD, standard deviation; Max, maximum; Min, minimum; m/s, metres per second; m, metre; s, second.

### Trunk kinematics

**FIGURE 1:**
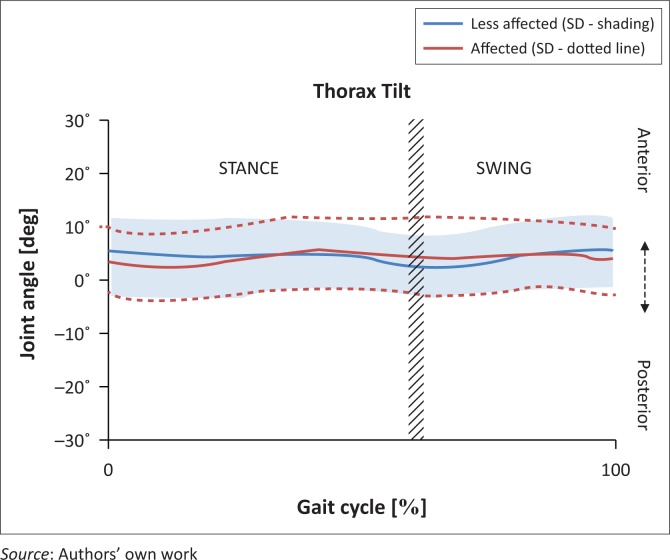
Trunk kinematics in the sagittal plane affected versus unaffected.

There was minimal trunk motion noted in the sagittal plane during the full gait cycle. The trunk largely remained anterior to neutral on both the affected (mean 4.28°, SD 0.87°) and unaffected sides (mean 4.33°, SD 0.90°). Figure 1 depicts a comparison between the affected and unaffected sides in degrees, with the red line representing the affected and the blue line depicting the unaffected side.

At initial contact, the trunk on the unaffected side was more anteriorly positioned (5.33°) than the affected side (3.56°), but this difference was not statistically significant. At foot off, there was a second difference noted with the affected side slightly more forward (1.77°). This finding reached statistical significance (Table 2).

**TABLE 2:** Mean (standard deviation) peak trunk angle during the full gait cycle, in the sagittal, coronal and transverse planes as well as at initial contact and foot off.

Thorax kinematics	Affected (degrees)	Less affected (degrees)	Mean difference (degrees)	Significance (*p* < 0.05)
**Full cycle**				
Sagittal	4.28 ± 0.87	4.33 ± 0.90	−0.05	0.500
Coronal	−2.17 ± 1.88	2.25 ± 1.93	−4.42	< 0.001*
Transverse	−3.54 ± 2.49	3.60 ± 2.61	−7.15	< 0.001*
**Initial contact**				
Sagittal	3.56 ± 5.98	5.33 ± 6.77	−1.77	0.988
Coronal	−2.01 ± 2.41	2.45 ± 3.19	−4.46	< 0.001*
Transverse	−6.63 ± 6.78	0.66 ± 6.16	−7.29	< 0.001*
**Foot off**				
Sagittal	4.35 ± 1.29	2.58 ± 1.41	1.77	< 0.001*
Coronal	0.26 ± 1.42	4.82 ± 1.35	−4.56	0.049*
Transverse	−2.45 ± 2.00	4.86 ± 1.57	7.31	0.013*

*Source*: Authors’ own work

* , statistical significance (*p* ≤ 0.05).

**FIGURE 2:**
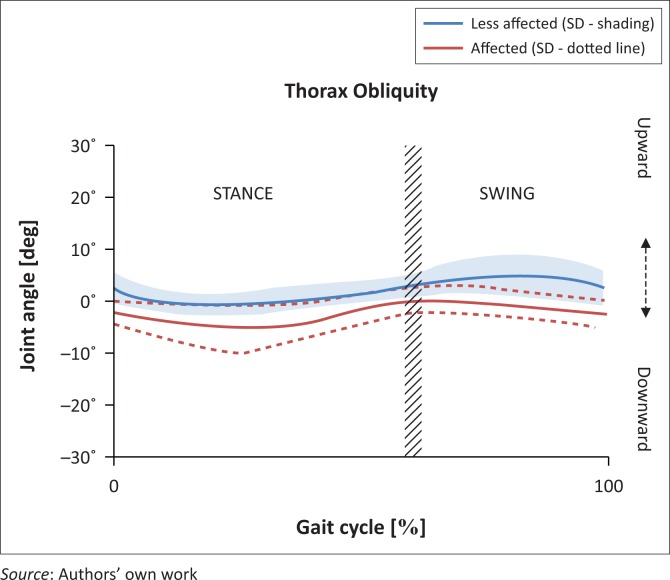
Trunk kinematics in the coronal plane affected versus unaffected.

Figure 2 illustrates the trunk kinematics in the coronal plane. The trunk remained fairly central throughout the gait cycle, although on the affected side it tended to move downwards (mean −2.17°, SD 1.88°), in contrast to the unaffected side (mean 2.25°, SD 1.93°).

At initial contact on the affected side, the trunk moved in a downward direction (mean −2.01°, SD 2.41°). In contrast, at initial contact on the unaffected side, the trunk tended to move upwards (mean 2.45°, SD 3.19°). At foot off on the affected side, the trunk was almost stationary, whereas on the unaffected side it moved upwards (mean 4.82°, SD 1.35°).

**FIGURE 3:**
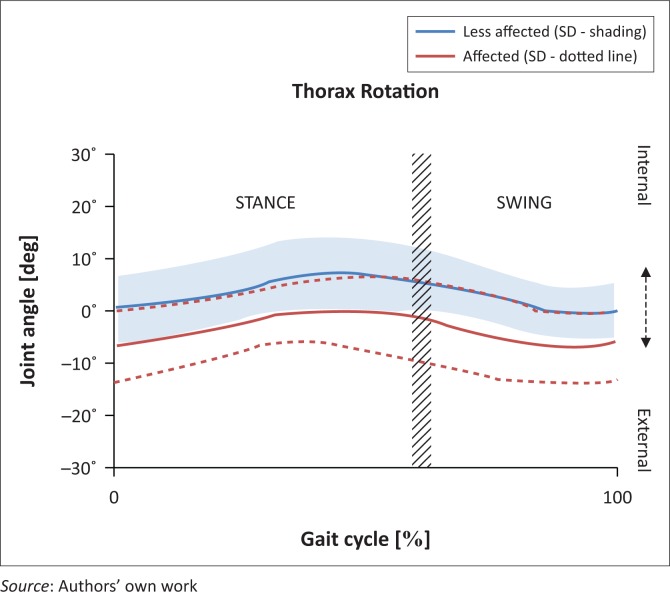
Trunk kinematics in the transverse plane affected versus unaffected.

In this plane, the trunk remained in a slightly backward rotated position during the full gait cycle (mean −3.54°, SD 2.49°) on the affected side, and obviously in a slightly forward rotated position (mean 3.60°, SD 2.61°) on the less affected side (Figure 3).

At initial contact on the affected side, the trunk rotated 6.63° (SD 6.78°) backwards as opposed to a fairly centrally positioned trunk on the unaffected side (mean 0.66°, SD 6.16°), indicating a statistically significant difference (*p* < 0.001). At foot off, the difference was also statistically significant, with the trunk rotated backwards (mean −2.45°, SD 2.00°) on the affected side and forward on the unaffected side (mean 4.86°, SD 1.57°).

## Ethical considerations

Ethical approval was granted by the Human Research Ethics Committee (HREC) of Stellenbosch University (reference number: S13/03/056) in July 2013 to conduct this observational descriptive study.

## Discussion

This study aimed to characterise key aspects of trunk motion during the full gait cycle of people with stroke using 3D kinematics for both the affected and unaffected sides. The secondary aims of the study included reporting of the spatiotemporal gait parameters of the sample.

The sample presented with characteristics commonly seen in the gait patterns of people with stroke, namely reduced cadence and walking speed (Shumway-Cook & Woollacott 2012). On average, 5 of the 17 participants in this study walked at ‘limited’ community speed (0.63 m/s) and the remaining 12 at community speed (1.03 m/s) (Schmid et al. 2007). Hemiparetic individuals tend to take shorter and wider steps at a slower gait speed compared to normal individuals (Hacmon et al. 2012). The participants in this study had a mean cadence of 101.63 steps per minute (SD 16.21) compared to 112.5 steps per minute for normal gait in adults (Shumway-Cook & Woollacott 2012).

### Trunk kinematics

Overall the trunk did not move through a large range of motion in the sagittal plane (anterior–posterior motion) and would be observed clinically as the trunk being held relatively still in a more anterior or forward tilted posture. Although some extension occurred, this movement never crossed neutrality (0° into extension). Normally there is not a large amplitude of movement, although there are clear flexion peaks at double support (i.e. initial contact) and extension peak at single support (i.e. midstance) (Krebs et al. 1992). The relatively rigid trunk position of this sample could be a compensatory attempt to maintain proximal stability, while the forward tilted position of the trunk may be used to aid forward propulsion by moving the centre of gravity forward. There was a statistically significant difference between the motion of the trunk during the stride of the affected and unaffected sides at foot off. However, this marginal difference could potentially have been attributed to measurement error, although the Vicon has demonstrated high accuracy and reliability (Ehara et al. 1995). It has been shown to have less than a 1.5° error (Richards 1999).

Normally the trunk moves side to side in the gait cycle (coronal plane) and aligns over each leg during its stance phase. This might be because of the need for support of the trunk during unilateral stance. It has been reported that the trunk moves towards the weight-bearing leg in normal gait at initial contact and then away from that side at terminal stance (Krebs et al. 1992; Whittle 2007). However, in our study, there was significant coronal asymmetry between the affected and unaffected sides during the full gait cycle, at initial contact, and at foot off, with the trunk moving downwards during stride of the affected side and upwards during stride of the unaffected side. This may be attributed to an altered strategy of the trunk to lengthen to support balance as the person commences and completes swing on the affected side, or a collapse of trunk stability during stance on the affected side.

During normal gait there is a forward swing of the pelvis on the side of the swinging leg, with either a counter-rotation of the trunk or the contralateral arm swinging forward leading to thoracic rotation (Lamoth et al. 2002). With an increase in walking speed, these reciprocal thoracic and pelvic rotations become more antiphase. However, in our study, mean trunk position during the gait cycle was slightly more forward than that of the pelvis. This infers that the participants were not accessing symmetrical counter-rotation and is supported by the clinical observation of a backward rotated trunk on the affected side.

Reducing gait asymmetry has been a goal as well as a measurement of success in gait re-education for people with stroke (Olney & Richards 1996). However, to date no relationship has been found between asymmetry and functional measures (e.g. gait speed) (Dodd & Morris 2003). Using the symmetry index described by Patterson et al. (2008), we found that the participants in our study did not exhibit spatiotemporal asymmetry and were all classified as limited or community walkers (Schmid et al. 2007). However, they presented with asymmetrical trunk kinematics. Balaban and Tok (2014) reported that while the normalisation of gait asymmetry is a common goal in post-stroke rehabilitation, this asymmetry may be an adaptation or compensation mechanism that allows the person to walk; therefore, symmetry should not be the goal of rehabilitation during the chronic phase after stroke. Griffin, Phdz and Mcbride (1995) suggested that aiming for symmetry in a stable body system (chronic stage of stroke) is not likely to have optimal performance as a consequence because an increase in the contribution of the affected side leads to asymmetry. They linked an increase in speed to optimal performance; however, an increase in speed in people with stroke will most likely lead to asymmetry. It is understandable to see asymmetry in a person with limbs having unequal capabilities (Griffin et al. 1995). It remains to be determined what the clinical and functional significance of truncal asymmetry actually is. Anecdotally, people with stroke wish to appear ‘normal’ and normal is viewed in a lay sense as symmetrical.

## Limitations of the study

The sample of this study were recruited from one setting, were a mixture of subacute and chronic, had received differing levels of rehabilitation experience and were all able to walk without the use of assistive devices. Therefore, the results of this study should not be generalised to the wider population of people with stroke and those with different or varying levels of function. This report focuses on the group data only, with an indication of individual variation provided by the standard deviations. It may be that with the expected heterogeneity in a stroke population, further individual analysis would yield more clinically meaningful information. Finally, the laboratory setting may have influenced the participants’ gait pattern as this does not emulate their natural environment.

### Clinical implications

In this study, trunk motion in people with stroke differed from that expected during normal gait. This took the form of reduced general motion with a tendency to lean forward, to the side and to rotate backwards on the affected side. These characteristics arguably reduce efficiency or increase energy (Patterson et al. 2010) and therefore require amelioration. However, this objective is not yet supported by evidence. Until such evidence appears, we would recommend that in the interests of patient-centred care if gait asymmetry is of concern to the people with stroke themselves, then it should be a goal in rehabilitation.

### Recommendations for future research

This study was a pilot study and provides preliminary quantified evidence that the trunk has asymmetric motion during gait after stroke in all three planes. Further investigation in a larger sample is required to determine if the trends noted can be replicated. A larger cohort will allow for subgroup analysis, such as determining the impact of the site and severity of lesion, different age groups, time since incident, comorbidities, varying functional levels, gender and BMI. The relationship between spatiotemporal parameters, trunk kinematics (asymmetries) and functional levels should be explored further.

## Conclusion

The aim of this study was to describe the kinematics of the trunk during gait of people with stroke. In summary, we found that the trunk remained relatively still during gait, but with significant asymmetries between the affected and unaffected sides. The participants were all functional walkers at a community level, yet still exhibited this asymmetry. It may be that rehabilitation needs to target the trunk as well as the limbs in hemiparetic gait.
